# Effects of Ionizing Radiation on DNA Methylation Patterns and Their Potential as Biomarkers

**DOI:** 10.3390/ijms26073342

**Published:** 2025-04-03

**Authors:** Lanfang Ma, Yu Zhang, Jie Xu, Yanan Yu, Pingkun Zhou, Xiuhua Liu, Hua Guan

**Affiliations:** 1 College of Life Sciences, Hebei University, Baoding 071002, China; 2656103961@aliyun.com; 2 Beijing Key Laboratory for Radiobiology, Department of Radiation Biology, Beijing Institute of Radiation Medicine, Beijing 100850, China; zhangyu18711452953@163.com (Y.Z.); 18391862300@163.com (J.X.); 2571733009@aliyun.com (Y.Y.); birm4th@163.com (P.Z.); 3 College of Public Health, University of South China, 28 West Changsheng Road, Hengyang 421000, China

**Keywords:** DNA methylation, ionizing radiation, biomarkers, radiation protection, epigenetics, radiation exposure assessment

## Abstract

DNA methylation is a common endogenous chemical modification in eukaryotic DNA, primarily involving the covalent attachment of a methyl group to the fifth carbon of cytosine residues, leading to the formation of 5-methylcytosine (5mC). This epigenetic modification plays a crucial role in gene expression regulation and genomic stability maintenance in eukaryotic systems. Ionizing radiation (IR) has been shown to induce changes in global DNA methylation patterns, which exhibit significant temporal stability. This stability makes DNA methylation profiles promising candidates for radiation-specific biomarkers. This review systematically examines the impact of IR on genome-wide DNA methylation landscapes and evaluates their potential as molecular indicators of radiation exposure. Advancing the knowledge of radiation-induced epigenetic modifications in radiobiology contributes to a deeper understanding of IR-driven epigenetic reprogramming and facilitates the development of novel molecular tools for the early detection and quantitative risk assessment of radiation exposure.

## 1. Introduction

Ionizing radiation (IR) induces various types of DNA damage, with double-strand breaks (DSBs) being among the most severe [1]. Among the established markers of DSBs, γ-H2AX, a phosphorylated form of histone H2AX, serves as a widely recognized biomarker due to its rapid formation and high sensitivity in detecting radiation exposure [2]. For several decades, conventional biological dosimetry techniques, such as the cytokinesis-block micronucleus (CBMN) assay and the dicentric chromosome (DC) assay, have been extensively utilized in radiation biodosimetry to assess actual exposure scenarios [3]. However, these methods present certain limitations when analyzing a large number of samples. Therefore, the development of novel biomarkers for ionizing radiation remains essential for advancing research and facilitating the rapid identification of radiation exposure in affected individuals.

Epigenetics functions as a dynamic regulatory mechanism within the genome, enabling adjustments in genome activity in response to environmental factors [4]. This mechanism also ensures the stable transmission of gene activity across cell division [5]. Epigenetic regulation primarily occurs through alterations in DNA methylation patterns, istone modifications, and changes in nucleosome positioning on DNA [6]. Increasing evidence suggests that epigenetics holds significant potential for identifying individual sensitivity to specific risk factors and predicting disease susceptibility [7]. Previous studies have indicated that ionizing radiation induces DNA hypomethylation in certain cancer types [8,9].

DNA methylation is not only an epigenetic phenomenon but also a potential biomarker for ionizing radiation. This process follows distinct patterns across different tissues and species [10] and undergoes changes with ageing [11]. As a crucial epigenetic mark, DNA methylation plays a fundamental role in regulating gene expression during embryonic development and throughout life. In the context of ionizing radiation, alterations in DNA methylation patterns can serve as indicators of cellular sensitivity to radiation exposure. This review aims to elucidate the DNA methylation changes induced by ionizing radiation, evaluate its potential as a biomarker, and analyze the dynamic variations in DNA methylation patterns in response to radiation exposure.

## 2. The Principle of DNA Methylation

### 2.1. Definition and Molecular Basis of DNA Methylation

As a stable epigenetic modification, DNA methylation is heritable across multiple cell divisions. Extensive research has focused on the establishment, maintenance, and removal of DNA methylation [12,13]. Methylation primarily occurs at CpG dinucleotides, where S-adenosyl methionine (SAM) donates a methyl group under the catalytic action of DNA methyltransferase (DNMT3), leading to the covalent attachment of a methyl group to the 5-carbon of cytosine, forming 5-methylcytosine (5-mC) [14,15].

**Figure 1:**
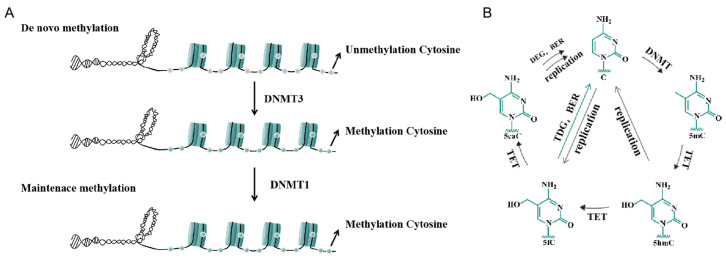
Principles of DNA methylation and demethylation. (**A**) Mammalian DNA methylation patterns. (**B**) The process of demethylation of mammalian DNA. TDG: thymine DNA glycosylase, BER: base-excision repair, DNMT: DNA methyltransferase.

Mammalian DNA methylation patterns are established and maintained by DNA methyltransferases (DNMTs), including DNMT1, DNMT3A, and DNMT3B, along with the accessory protein DNMT3L. Research indicates that DNMT3A exhibits greater activity on unmethylated DNA substrates compared to semi-methylated DNA [16], and DNMT3A [17], DNMT3B, and DNMT3L play essential roles in the initial establishment of DNA methylation patterns. DNMT1, which has a higher affinity for semi-methylated DNA than for unmethylated DNA [16,18], ensures the faithful maintenance of established methylation patterns [19] (Figure 1A).

DNA demethylation involves the replacement of 5mC with cytosine [20] and occurs through both passive and active mechanisms in mammalian cells. Passive demethylation takes place during DNA replication, as the newly synthesized DNA strand remains initially unmethylated. Under normal conditions, DNA methyltransferases restore methylation, but when these maintenance mechanisms are inhibited or dysfunctional, successive replication cycles result in the progressive loss of methylation [21]. However, this model exhibits low efficiency. In active demethylation, 5mC undergoes enzymatic oxidation by TET enzymes, converting into 5-hydroxymethylcytosine (5hmC), 5-formylcytosine (5fC), and 5-carboxycytosine (5caC). The latter two intermediates are subsequently excised through thymine DNA glycosylase (TDG) and base-excision repair (BER), ultimately leading to the removal of the methyl group and the completion of the demethylation process (Figure 1B) [22,23].

### 2.2. Role of DNA Methylation in Gene Expression Regulation

The gene structure consists of the promoter, exon, intron, terminator, 5′ untranslated region (5′ UTR), and 3′ untranslated region (3′ UTR). DNA methylation in these regions influences gene expression and function in distinct ways.

#### 2.2.1. DNA Methylation of Gene Promoters Regulates Gene Expression

In most cases, DNA methylation in promoter regions of human genes results in decreased expression or complete gene silencing [24]. DNA methylation plays a critical role in regulating gene transcription by ensuring accurate gene expression and preventing the activation of false promoters or aberrant splicing events, thereby maintaining transcriptional fidelity [25,26]. Methylated cytosine (5mC) can directly suppress transcription by obstructing the binding of transcriptional activators to their respective DNA recognition sequences. Alternatively, methyl-CpG-binding domain proteins (MBDs) can mediate gene silencing by recruiting co-repressors, leading to an inhibited state of methylated DNA [27,28].

In ovarian endometriosis (OE), Maekawa et al. reported that methylation levels in the T-DMR1 and T-DMR2 regions of the ESR1 gene were significantly elevated compared to those in normal endometrial tissue (EE) [29]. The CCCTC-binding factor (CTCF) has been shown to interact with the ESR1 promoter in MCF-7 cells, potentially affecting ESR1 transcription [30]. Furthermore, extensive methylation of the FOXP3 promoter region suppresses FOXP3 expression, which has been linked to the progression of hepatocellular carcinoma [31]. Increased Foxp3 DNA methylation levels have also been associated with exposure to Bisphenol A (BPA), with Foxp3 DNA methylation identified as a key molecular mechanism underlying BPA-induced immune dysfunction [32].

#### 2.2.2. DNA Methylation of Gene Introns Regulates Gene Expression

Although intron methylation is less significant in gene expression regulation compared to promoter region methylation, it still plays a crucial role in certain biological processes.

In patients with Parkinson’s disease (PD), particularly those with the PD-GBA1 subtype, alterations in DNA methylation at specific CpG sites within the intron 1 region of the alpha-synuclein (SNCA) gene have been observed, with more pronounced changes in the frontal cortex. Additionally, epigenetic differences exist between PD-GBA1 and idiopathic PD [33]. In breast cancer samples, reduced intron methylation has been associated with increased mRNA expression, whereas higher intron methylation levels correlate with greater mRNA retention [34]. Furthermore, a decline in DNA methylation within intron 1 of the TREM2 gene in peripheral white blood cells of Alzheimer’s disease (AD) patients has been linked to elevated TREM2 mRNA expression, indicating its potential as a biomarker for AD [35].

#### 2.2.3. DNA Methylation of Gene Exons Regulates Gene Expression

In the genome, hypermethylation has been associated with increased gene expression [36]. DNA methylation regulates alternative splicing by directly modifying the methylation levels of specific exons and by influencing the structure of chromatin and histone modifications [37]. In breast cancer patients under hypoxic conditions, alterations in exon DNA methylation levels of specific genes, such as DHX32 and BICD2, have shown significant correlations with alternative splicing events and gene expression levels. The methylation status of these exons has also been closely linked to patient survival, highlighting the potential regulatory role of exon DNA methylation in breast cancer progression and prognosis within a hypoxic environment [38]. Additionally, changes in the DNA methylation of SOCS3 exon 2 in visceral adipose tissue from patients with gestational diabetes mellitus have been associated with increased mRNA expression [39].

DNA methylation regulates gene expression through multiple mechanisms and plays a critical role in disease onset and progression by influencing gene transcription, splicing, and expression, making it a potential biomarker for diagnosis and therapeutic applications.

## 3. DNA Methylation Markers: Unique Advantages in Clinical Applications

DNA methylation markers show high sensitivity and specificity in the early screening of a variety of tumors. In multiple cancer early-detection studies, cfdna-based DNA methylation markers were sensitive and specific for colorectal cancer [40], gastric cancer [41], esophageal cancer, liver cancer, lung cancer, ovarian cancer, and pancreatic cancer [42]. In gynecologic genital tract tumors, the cfDNA methylation marker identified early ovarian cancer patients with 90.57% specificity and 85.37% sensitivity compared with the conventional marker, CA125, which had only 56.10% sensitivity and 64.15% specificity [43]. The methylation marker of the Septin9 gene (mSEPT9) [44] is a specific marker used for the detection of colorectal cancer, and it has been widely used in clinical diagnosis. Moreover, the RASSF1A gene promoter is methylated in up to 88% of lung cancer cases and exhibits almost no methylation in normal peripheral tissues, so detecting the methylation status of the RASSF1A gene can serve as a sensitive and specific marker for the early detection of lung cancer [45]. The methylation levels of five genes, H2AFY, CTSA, LTC4S, IL5RA, and RB1, were significantly associated with the clinical response before treatment, and may serve as potential markers of radiotherapy response in breast cancer [46].

Samples from sputum, plasma, serum, or urine can be obtained in a non-invasive manner. Endometrial cancer is diagnosed by testing for DNA methylation markers (GHSR, SST, ZIC1) in urine [44]. DNA methylation markers are tested for the diagnosis of oral cancer by using non-invasive (saliva or oral rinse) or minimally invasive (oral brush or blood) samples [47].

Certain DNA methylation anomalies emerge at the very beginning of tumor formation, and by identifying methylation markers tied to tumor development and gauging the risk of progression, they can aid in the early diagnosis of cancer. For instance, the RNF180/Septin9 [41] gene methylation test is applicable for early screening of gastric cancer. Moreover, combining the detection of RNF180 and Septin9 gene methylation with traditional tumor markers can significantly enhance the sensitivity of gastric cancer screening.

DNA methylation markers exhibit remarkable sensitivity and specificity in the early detection of a wide range of tumors. Through the identification of non-invasive DNA methylation markers in various samples, the accuracy of early cancer diagnosis can be significantly enhanced, thereby providing robust evidence for early intervention and the targeted treatment of tumors.

## 4. Effects of Ionizing Radiation on DNA Methylation

### Oxidative Stress and DNA Damage

Ionizing radiation reduces overall DNA methylation levels, leading to an increase in DNA strand breaks and enhanced recombination activity. The decrease in methylation levels in irradiated tissue cells may be associated with their ability to repair damage. During the repair process, DNA polymerase incorporates cytosine instead of methylated cytosine. As a result, the induction of DNA damage and the activation of repair and recombination mechanisms may contribute to hypomethylation. Additionally, exposure to ionizing radiation inhibits DNA methylation, which is linked to the activation of DNA modification processes [48].

Ionizing radiation induces ionization and excitation of water molecules in biological systems, leading to the generation of highly reactive oxygen species (ROS) free radicals [49]. ROS-mediated DNA damage reduces gene methylation at the injury site, thereby increasing gene expression [50]. Although DNA repair enzymes efficiently restore damaged DNA, studies suggest that DNA damage response genes, such as GADD45A and nuclear protein 95 (Np95), selectively methylate specific DNA regions to facilitate repair [51]. GADD45A exhibits strong binding affinity to hemimethylated DNA intermediates, initiating the recruitment of Np95 [52]. Subsequently, Np95 directs histone H3 methyltransferase to the damaged DNA site and facilitates the recruitment of de novo methyltransferases Dnmt3a and Dnmt3b, resulting in DNA methylation. Moreover, epigenetic methylation alterations in the genome are influenced by ROS, which induces DNA breaks that are later repaired and methylated by DNA methyltransferases (DNMTs).

Ionizing radiation induces genome-wide DNA hypomethylation (Table 1). Koturbash et al. [53] reported significant alterations in DNA methylation patterns in the thymus of C57/BL6J mice after acute or 10-day subchronic X-ray exposure at 5 Gy, leading to persistent genome-wide DNA hypomethylation. Pogribny et al. [54] observed radiation-induced whole-genome DNA hypomethylation in liver and spleen tissues of female and male C57/BL6J mice six hours after acute whole-body X-ray exposure at doses ranging from 0.5 to 5 Gy. The hypomethylation effect in male mice was dose-dependent, whereas chronic exposure to 5 Gy did not induce significant changes. Wang et al. [55] also confirmed that blood genome hypomethylation occurred in male BALB/c mice following acute exposure to 0.5 Gy X-ray or chronic exposure to 0.05 Gy accumulated over 10 days.

Akifumi Nakata et al. [56] investigated the effects of chronic low-dose radiation (LDR) and acute high-dose radiation (HDR) on DNA methylation patterns and Izumo1 and Izumo1r gene expression in mouse testis and ovaries. The LDR group retained DNA methylation levels, whereas the HDR group exhibited a progressive decline with age. Izumo1 expression recovered after LDR irradiation but remained low following HDR irradiation. Izumo1r expression was low in both LDR and HDR groups, suggesting that varying radiation doses may influence germ cell development and fertility through alterations in DNA methylation. Furthermore, radiation-induced methylation of the CpG site in the p16 promoter in thymic lymphoma samples has been implicated in reduced gene expression in post-radiation thymic lymphomas [57].

Radiation-induced changes in genomic DNA methylation are dose-dependent, sex-specific, and tissue-specific, with these alterations being persistent [54]. Additionally, exposure to low doses of ionizing radiation has been associated with global DNA hypomethylation [58]. According to the above research, the feasibility of DNA methylation as a biomarker of ionizing radiation is as follows.

The DNA methylation changes induced by ionizing radiation exhibit tissue specificity and relative stability, making them promising candidates as biomarkers. For instance, studies have shown that distinct tissues display unique methylation profiles following radiation exposure, and these profiles tend to persist over time [54].

Maturity of detection technology: At present, the detection technology of DNA methylation is quite mature, and it can detect the methylation status with high sensitivity and high specificity. Methylation-specific PCR (MSP) [47] and its derivative techniques can be used to detect free DNA (cfDNA) in body fluids (e.g., blood [59], saliva [60], urine [61]) for non-invasive testing.

Advantages over traditional biomarkers: DNA methylation markers show higher sensitivity and specificity in early detection than traditional biomarkers. The specificity of DNA methylation markers can reach over 90% in the early detection of some cancers.

Reflecting the long-term effects of radiation exposure: DNA methylation changes can not only be detected in a short term after radiation exposure but also for a long time, thus reflecting the long-term effects of radiation exposure [62]. This allows DNA methylation markers to be used to assess the health risks after radiation exposure.

## 5. Biomarkers of Ionizing Radiation

The ideal biomarker for ionizing radiation should exhibit a high sensitivity to dose variations, be novel, and be easy to utilize. These biomarkers must remain stable post-exposure and enable repeated assays in a minimally invasive manner [63]. The currently available categories of ionizing radiation biomarkers are discussed below.

### 5.1. Classical Biomarkers of Ionizing Radiation—γ-H2AX

DNA damage induced by ionizing radiation primarily manifests as DNA double-strand breaks (DSBs). When DSBs occur, histone H2AX undergoes phosphorylation at serine 139 (γ-H2AX) at the break site [64,65]. The number of γ-H2AX foci reaches its peak about 30 min after ionizing radiation exposure, decreases significantly within 24 h, and returns to baseline levels within a few days, depending on the radiation dose. The number of γ-H2AX foci is also dose-dependent [66].

As a biomarker, γ-H2AX offers advantages such as high sensitivity, specificity, and ease of detection. However, certain limitations exist, including its transient nature. Since γ-H2AX formation and persistence are dynamic processes, the signal weakens or disappears as DNA damage is repaired, and improper detection timing may lead to misinterpretation. Additionally, background interference from inherent cellular signals during detection can affect the accuracy. Therefore, further research on alternative ionizing radiation biomarkers is necessary to enhance the rapid diagnosis of radiation-induced injury and mortality [67].

### 5.2. Alternative Biomarkers-RNA

RNA molecules play essential roles in cellular functions, and post-transcriptional modifications are crucial for regulating RNA stability, transport, processing, and gene expression. These modifications contribute to the fine-tuning of the cellular transcriptome, ensuring accurate and efficient protein synthesis [68].

#### 5.2.1. RNA Biomarkers

Ionizing radiation (IR) induces distinct alterations in miRNA expression within cells. Templin et al. [69] found that the miRNA expression profiles of human peripheral blood lymphocytes following γ-ray irradiation varied between normal and microgravity conditions, with fewer miRNAs responding to radiation under microgravity. Differentially expressed miRNAs have the potential to serve as biomarkers for radiation exposure.

#### 5.2.2. mRNA Biomarkers

The analysis of mRNA transcripts responsive to radiation revealed that Ncoa4, Atel, and Fgf22 exhibited dose-dependent reactivity in RNA m^6^A levels, suggesting their potential as biomarkers for radiation exposure [70]. Additionally, genes such as SELE, ASPM, and BRCA2 displayed significant, dose-dependent expression changes in endothelial cells following X-ray irradiation, indicating their possible utility for assessing radiation dose effects [71]. Furthermore, the expression of genes including DDB2, PCNA, GADD45A, SESN1, RRM2B, KCNN4, IFI30, and PTPRO increased in humans but decreased in mice post-X-ray irradiation, highlighting species-specific gene expression variations and their potential as biomarkers for human responses to radiation [72].

RNA molecules are extensively studied as biomarkers of ionizing radiation due to their expression changes following radiation exposure. However, current research on DNA methylation biomarkers primarily focuses on clinical disease diagnosis, with limited application to ionizing radiation. Given the high sensitivity of peripheral blood lymphocytes to ionizing radiation, further investigation into DNA methylation markers for radiation exposure assessment is necessary.

### 5.3. DNA Methylation Biomarkers

Ionizing radiation induces alterations in genome-wide DNA methylation levels, including both global hypomethylation and hypermethylation in specific gene regions, which may be closely associated with the biological effects of radiation. Magy Sallam et al. [73] analyzed DNA methylation in heart-exposed rats and breast cancer (BC) patients undergoing radiation therapy and found that high-dose X-ray exposure resulted in long-term methylation alterations and differential expression of heart-related genes, including *SLMAP* and *E2F6*, over a period of up to seven months. These modifications were linked to decreased cardiac function and were validated in patient samples, suggesting that systemic radiation exposure can be detected using DNA methylation as a biomarker.

Jihye Park et al. [74] found that even low doses of ionizing radiation (0.1 Gy and 0.5 Gy) induce genome-wide DNA methylation changes in normal endothelial cells (HAEC and HCAEC). Genes such as PGRMC1, UNC119B, RERE, and FNDC3B were identified as potential DNA methylation biomarkers for assessing cardiovascular risk following low-dose radiation exposure. Additionally, Kuzmina et al. [75] investigated long-term hypermethylation in leukocyte genes in human blood post-radiation exposure and found a significantly higher frequency of CpG island hypermethylation in the promoter regions of *GSTP1*, *TP53*, and *SOD3* in the exposed group compared to the control group, indicating that radiation exposure contributes to gene hypermethylation.

DNA methylation markers offer substantial advantages in detecting ionizing radiation exposure and can effectively address the limitations of traditional biomarkers. These advantages are detailed in the following subsections.

#### 5.3.1. High Specificity and Stability

DNA methylation changes exhibit high tissue specificity and relative stability [74]. Unlike traditional biomarkers (such as chromosome aberrations and protein markers), DNA methylation markers remain stable for extended periods following radiation exposure, without rapid degradation due to cellular metabolism or environmental factors [76]. This stability enables DNA methylation markers to more accurately reflect the long-term effects of radiation exposure. Traditional markers, like γ-H2AX [77], can rapidly indicate DNA damage but are short-lived, making them unsuitable for long-term exposure evaluation. DNA methylation markers, however, can compensate for this deficiency and provide a reliable basis for long-term radiation exposure monitoring [78].

#### 5.3.2. Non-Invasive Testing

DNA methylation markers can be detected non-invasively through free DNA (cfDNA) in body fluids such as blood, saliva, and urine. This detection method not only minimizes patient discomfort but also enhances the convenience and acceptability of testing. Traditional markers, such as chromosomal aberrations, often require complex and invasive procedures like cell culture or tissue biopsy. In contrast, the non-invasive detection of DNA methylation markers offers the potential for mass screening and long-term monitoring.

#### 5.3.3. Multi-Dimensional Biological Significance

DNA methylation changes can influence multiple biological processes including gene expression, cell cycle regulation, and apoptosis. By analyzing the DNA methylation status, one can gain a comprehensive understanding of radiation’s impact on cells and tissues, rather than merely observing the direct result of DNA damage. While traditional markers (such as cytokines and metabolites) can reflect cellular stress responses, they do not directly reveal changes at the gene regulation level. DNA methylation markers, however, provide deeper biological insights, helping researchers and clinicians better understand cellular responses to radiation exposure.

#### 5.3.4. Early Detection and Risk Assessment

DNA methylation markers can reflect early changes following radiation exposure, and the detection sensitivity and specificity can be enhanced by combining multiple markers. For example, methylation detection of the RNF180/Septin9 gene has shown significant advantages in the early screening of gastric cancer. Traditional markers, like gamma-H2AX, while capable of rapidly detecting DNA damage, have limited sensitivity and specificity in early cancer screening. DNA methylation markers can detect cfDNA in body fluids to achieve early cancer screening and provide a basis for early intervention and treatment.

#### 5.3.5. Potential for Personalized Medicine

DNA methylation markers can assess an individual’s susceptibility to radiation, thereby supporting personalized medicine. For instance, testing the methylation status of specific genes can predict a patient’s response to radiation therapy and help develop more precise treatment plans. Traditional markers, such as cytokines, can reflect cellular stress responses but cannot provide individualized predictive information. DNA methylation markers, by detecting an individual’s gene methylation status, offer strong support for personalized medicine.

In summary, DNA methylation markers are not only highly specific and stable but also enable early screening and long-term monitoring through non-invasive testing. Their multi-dimensional biological significance at the gene regulation level allows them to fully reflect the cellular response after radiation exposure and provides support for personalized medicine. Therefore, DNA methylation markers can effectively complement the shortcomings of traditional biomarkers and offer a novel approach for the detection and evaluation of ionizing radiation exposure.

## 6. Conclusions and Perspectives

This review has comprehensively explored the intricate relationship between ionizing radiation (IR) and DNA methylation, highlighting the significant potential of DNA methylation as a biomarker for IR exposure. The findings underscore several critical aspects that warrant further exploration and application in both research and clinical settings.

### 6.1. The Complex Interaction Between IR and DNA Methylation

Ionizing radiation induces a wide range of DNA damage, with double-strand breaks (DSBs) being particularly severe. The formation of γ-H2AX, a well-established biomarker for DSBs, reflects the immediate cellular response to radiation-induced DNA damage. However, the long-term effects of IR exposure extend beyond the initial DNA breaks. Epigenetic modifications, particularly DNA methylation, play a crucial role in shaping the cellular response to IR. As demonstrated in numerous studies, IR exposure can lead to both global hypomethylation and localized hypermethylation, affecting gene expression, cell cycle regulation, and apoptosis. These changes are not only tissue-specific but also exhibit relative stability over time, making them ideal candidates for long-term biomarkers of IR exposure.

### 6.2. DNA Methylation as a Biomarker for Ionizing Radiation Exposure

The potential of DNA methylation as a biomarker for IR exposure is supported by several key advantages. (1) High specificity and stability: Unlike traditional biomarkers, such as γ-H2AX, which are transient and degrade rapidly, DNA methylation changes remain stable over extended periods. This stability allows for accurate long-term monitoring of IR exposure, even months or years after the initial event. (2) Non-invasive detection: DNA methylation can be assessed through free DNA (cfDNA) in body fluids like blood, saliva, and urine. This non-invasive approach minimizes patient discomfort and enhances the feasibility of large-scale screening and long-term monitoring. (3) Multi-dimensional biological significance: DNA methylation changes provide insights into multiple biological processes, including gene expression and cell cycle regulation. This comprehensive view of cellular responses to IR exposure offers deeper biological insights compared to traditional markers. (4) Early detection and risk assessment: DNA methylation markers can detect early changes following IR exposure, with high sensitivity and specificity. For example, the methylation status of genes like RNF180 and Septin9 has shown significant promise in early cancer screening. (5) Personalized medicine: DNA methylation markers can assess an individual’s susceptibility to IR, supporting personalized treatment plans. By predicting patient responses to radiation therapy, these markers can enhance the precision of medical interventions.

### 6.3. Addressing the Limitations of Traditional Biomarkers

Traditional biomarkers for IR exposure, such as γ-H2AX and chromosomal aberrations, have been instrumental in radiation biodosimetry. However, they present certain limitations, including transient signals, complex detection procedures, and limited specificity. DNA methylation markers address these shortcomings by offering a stable, non-invasive, and highly specific alternative. Their ability to reflect long-term effects and provide deeper biological insights makes them a valuable addition to the toolkit of radiation exposure assessment.

### 6.4. Future Directions and Clinical Implications

The potential of DNA methylation as a biomarker for infrared exposure opens up new avenues for research and clinical applications. Future research should focus on (1) validation and standardization: further validation of specific DNA methylation markers in different populations and exposure scenarios is needed to establish standardization protocols. (2) Integration with other biomarkers: Combining DNA methylation markers with existing biomarkers, such as gamma-H2AX, can improve the accuracy and comprehensiveness of IR exposure assessments. (3) Clinical applications: explore the application of DNA methylation markers in personalized medicine, especially in predicting patient response to radiation therapy and optimizing treatment plans.

In conclusion, the relationship between ionizing radiation and DNA methylation is complex and multifaceted. DNA methylation markers offer significant advantages over traditional biomarkers, providing a stable, non-invasive, and highly specific means of assessing IR exposure. Their potential for early detection, risk assessment, and personalized medicine underscores their importance in advancing our understanding and management of radiation exposure. Future research should continue to explore and validate the application of DNA methylation markers in both research and clinical settings.

## Figures and Tables

**Table 1:** Effect of ionizing radiation on global DNA methylation.

Subjects	Type of radition	Dose of radition	Conclusion
CHO K-1 HeLa S-3, C-i1300N1E-115, V79A03	Acute Radition	10 Gy	Genomic DNA methylation levels decreased in a dose-dependent manner, with genome-wide demethylation being most pronounced after 48 h
C57/BL6J mice	Acute Radition	4, 7, 10 Gy	Genome-wide DNA hypomethylation of stem cells was observed after 8, 24, 48, and 72 h
Thymus of C57/BL6J mice	Acute Radition	5 Gy	Genome-wide DNA hypomethylation, persistent
Liver and spleen of C57/BL6J mice	Acute Radition	0.5, 5 Gy	Genome-wide DNA hypomethylation, Dose dependence, especially in male mice
Blood from BALB/c mice	Acute/Chronic Radition	0.5 Gy, 0.05 Gy × 10	Genomic hypomethylation was observed after acute and chronic exposures
mice testis and ovary	Acute/Chronic Radition	4 Gy (0.47 Gy/min) 4 Gy (0.1 mGymin)	LDR maintained methylation level, while HDR decreased. Izumo1 expression recovered after LDR and remained low after HDR
Thymus of BALB/c mice	Chronic Radition	7 Gy (1.75 Gy × 4)	Methylation of CpG sites in the p16 promoter would reduce its expression in post-radiation thymic lymphoma

## Data Availability

Not applicable.
